# A macroreentrant biatrial tachycardia via the posterior interatrial connection emerged after extensive catheter ablation for paroxysmal atrial fibrillation

**DOI:** 10.1016/j.hrcr.2022.10.009

**Published:** 2022-10-08

**Authors:** Yasunori Kanzaki, Itsuro Morishima, Hiroyuki Miyazawa, Kazuki Shimojo

**Affiliations:** Department of Cardiology, Ogaki Municipal Hospital, Ogaki, Japan

**Keywords:** Atrial fibrillation, Biatrial tachycardia, Catheter ablation, High-resolution mapping, Posterior interatrial connection, Reentrant circuit

**Key Teaching Points**

• A patient without open heart surgery developed a macroreentrant biatrial tachycardia via the posterior interatrial connection.
• Previous radiofrequency catheter ablation of the atrial septum may have slowed conduction in the intra-atrial septum, which allowed the signal to travel in a biatrial circuit via the posterior interatrial connection.
• High-resolution mapping identified the complex biatrial tachycardia circuit and radiofrequency application at the right atrial breakout of the posterior interatrial connection terminated the tachycardia.

## Introduction

Recent electrophysiological studies have demonstrated the existence of an epicardial connection via posterior interatrial fibers, which connect the antrum of the right pulmonary veins and the right atrium (RA).^1^ This connection represents a potential mechanism for the interference of circumferential antral pulmonary vein isolation.^2^ The posterior interatrial connection has also been reported as a critical limb of biatrial reentrant circuits in a rare condition with interatrial septal incisions after mitral valve surgery.^3^ Here, we describe a case of complex macroreentrant biatrial tachycardia associated with a posterior interatrial connection, which developed after pulmonary vein isolation and atrial septum ablation for non–pulmonary vein foci.

## Case report

**Figure 1:**
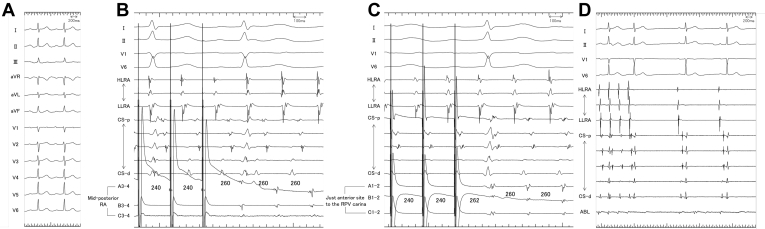
**A:** Twelve-lead electrocardiogram during atrial tachycardia with a cycle length of 260 ms. Positive/negative P wave in lead V_1_ and positive P wave in inferior leads were present. **B, C:** Atrial overdrive pacing at exit (**B**, the mid-posterior right atrium) and the entrance (**C**, just anterior to the isolated right pulmonary vein carina) of the posterior interatrial connection could entrain the atrial tachycardia, and the postpacing intervals were in accordance with the cycle length of the atrial tachycardia at both sites. **D:** Surface and intracardiac electrograms after radiofrequency application at the successful site. The tachycardia cycle length gradually increased and finally terminated. ABL = ablation catheter; CS = coronary sinus; d = distal; HLRA = high lateral right atrium; LLRA = low lateral right atrium; p = proximal; RA = right atrium; RPV = right pulmonary vein.

**Figure 2:**
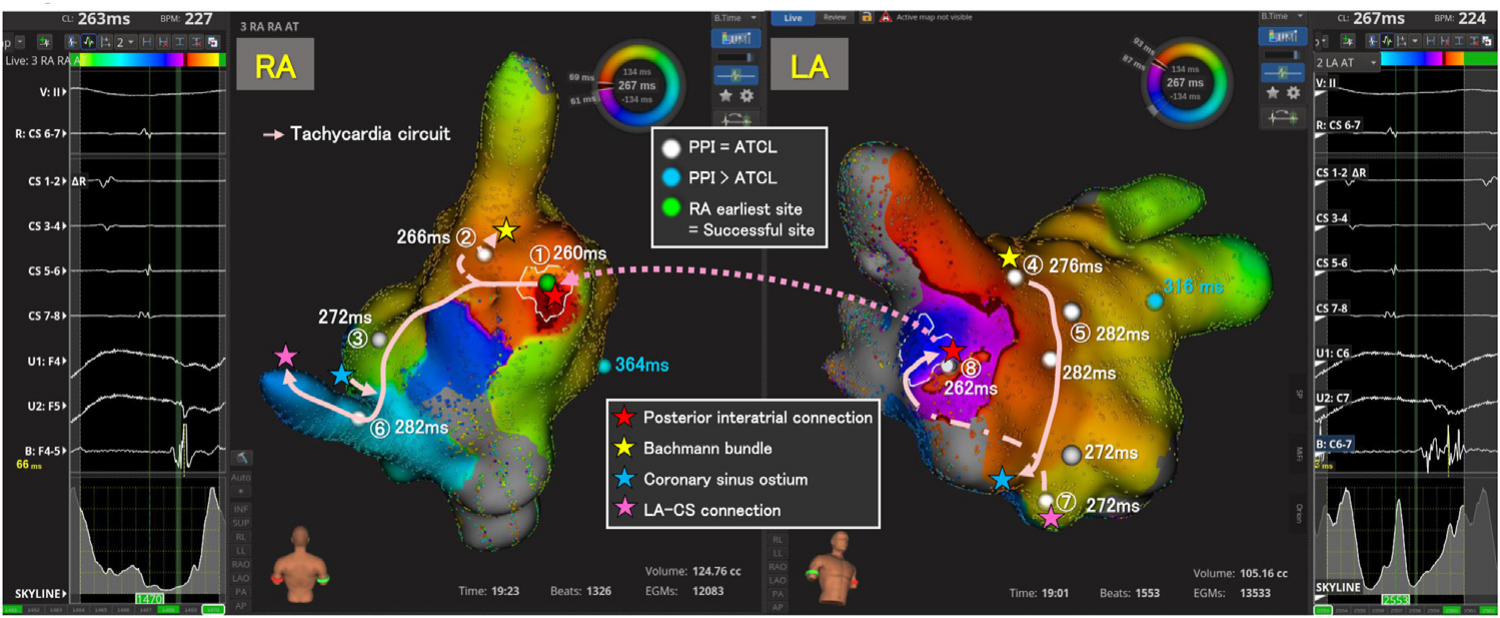
High-resolution activation map of the atrial tachycardia (AT). The cardiac tracings represent the potentials obtained at the entrance (right panel) and exit (left panel) sites of the posterior interatrial connection. Mapping the right atrium (RA) demonstrates a centrifugal activation pattern, with the earliest site at the mid-posterior RA (①). The signal traversed the RA anteroseptum (②) and mid-septum (③) and was conducted to just outside the anterior right superior pulmonary vein isolation line (④) through the Bachmann bundle. It then moved down along the anterior left atrium (LA) (⑤) to the coronary sinus ostium (⑥), where it combined with another signal that had come down along the RA septum. This combined signal traveled through the posterior LA (⑦) via the LA–coronary sinus connection to the site just anterior to the isolated right pulmonary vein carina, entering the posterior interatrial connection (⑧), and then broke out to the mid-posterior RA (①) through the connection. There was a conduction block between sites ④ and ⑧. The atrial tachycardia was entrained, and the postpacing intervals were in accordance with the atrial tachycardia cycle length at each point, except at the low lateral RA and LA appendage.

A 55-year-old man developed atrial tachycardia after multiple sessions of catheter ablation for paroxysmal atrial fibrillation. The heart was structurally normal, and the left atrium (LA) was not enlarged. The patient had undergone pulmonary vein isolation with cryoballoon and additional radiofrequency ablation, including LA posterior wall isolation, cavotricuspid isthmus ablation, and non–pulmonary vein foci ablation in the right and left atrial septa, coronary sinus ostium, and the LA bottom. The P-wave morphology during the tachycardia showed positive/negative in lead V_1_ and positive in inferior leads with a cycle length of 260 ms (Figure 1A). High-resolution activation mapping demonstrated a centrifugal activation pattern, with the earliest site at the mid-posterior RA (Figure 2). The atrial tachycardia (AT) was entrained, and the postpacing intervals were in accordance with the AT cycle length at the earliest activation site (Figure 1B), RA anteroseptum, RA mid-septum, and ostium of the coronary sinus, but not at the low lateral RA. LA activation began outside the anterior right superior pulmonary vein isolation line, with the postpacing intervals in accordance with the AT cycle length at this site, the mid-anterior LA, posterior bottom of the LA, and just anterior to the isolated right pulmonary vein carina (Figure 1C), but not at the LA appendage. The signal in the LA moved down along its anterior to the ostium of the coronary sinus, where it combined with another signal that came down along the RA septum. This combined signal traveled through the LA posteriorly, via the LA–coronary sinus connection, to just anterior to the isolated right pulmonary vein carina and then broke out to the mid-posterior RA (Supplemental Movie). These findings suggested biatrial tachycardia, with one limb formed by the posterior interatrial connection and the other limb formed by the Bachmann bundle and coronary sinus (Figure 2). The conduction velocity of the posterior interatrial connection during the tachycardia was calculated as approximately 0.9 m/s. A single radiofrequency application at the earliest site of the RA terminated the atrial tachycardia (Figure 1D). The patient has been free from either atrial fibrillation or AT without any antiarrhythmic medications for the subsequent 12 months.

## Discussion

Previous studies have suggested that a posterior interatrial connection bridges the antrum of the right pulmonary vein and mid-posterior RA.^1^ In some cases, pulmonary vein isolation fails, and additional radiofrequency ablation at the antrum of the right pulmonary vein is required for this epicardial connection.^2^ In our case, the right pulmonary veins were individually isolated using a cryoballoon, and the anterior antrum of the right pulmonary vein was outside of the isolation line. As a result, the posterior interatrial connection remained outside the right pulmonary vein isolation line.

The P wave is useful to localize the origin of focal AT.^4^ Meanwhile, the P-wave morphology during macroreentrant AT is determined by the activation pattern within the dominant atrium with more muscle volume.^5^ In the present case, the AT showed P-wave morphology similar to the focal atrial tachycardia arising from the RA breakout site. This might be explained by the previous extensive LA ablation including pulmonary veins, posterior wall, septum, and bottom. There may have been a significant decrease in recruitable LA muscle volume, making the RA the dominant atrium with more muscle volume.

The posterior interatrial connection could also act as a critical limb of biatrial reentrant circuits when combined with other interatrial connections, such as the Bachmann bundle, fossa ovalis, and ostium of the coronary sinus, in patients with interatrial septal incision after mitral valve surgery.^3^ However, to our best knowledge, macroreentrant biatrial tachycardia associated with the posterior interatrial connection has never been reported in patients without prior open heart surgery. In the present case, conduction slowdown was not evident in the posterior interatrial connection during tachycardia. The conduction disturbance caused by the previous ablation in the atrial septum might be involved in tachycardia development. There was a noticeable conduction delay at the posterior septum of the RA and ostium of the coronary sinus, lengthening the propagation time from RA posterior interatrial connection breakout site to the coronary sinus. On the other hand, the LA activation following the breakout from the Bachmann bundle moved down along the LA anteriorly but could not directly contribute to the posterior interatrial connections owing to conduction delay at the right anterior LA. The signal traveled to the ostium of the coronary sinus and combined with another signal, which propagated through the coronary sinus slowly. The multiple conduction disturbances present in both interatrial septa might have been responsible for the development of the complex macroreentrant biatrial tachycardia. Additionally, the isolation of the LA posterior wall as well as the cavotricuspid isthmus line might have been involved in AT occurrence by limiting the wavefronts coming from both lateral regions to the atrial septum. These iatrogenic aspects of tachycardia should be noted.

## Conclusion

Macroreentrant biatrial tachycardia associated with the posterior interatrial connection might develop after extensive catheter ablation for atrial fibrillation management. High-resolution mapping is useful to delineate the complex tachycardia circuit. The right atrial breakout of the posterior interatrial connection may be the optimal target of catheter ablation for tachycardia management.

## Supplementary data

Supplemental Movie Legend A high-resolution activation map of the atrial tachycardia demonstrates a centrifugal activation pattern in the right atrium (RA), with the earliest site at the mid-posterior RA. The signal traversed the RA at the antero-septum and mid-septum and was conducted to just outside the anterior right superior pulmonary vein isolation line through the Bachmann bundle. It then moved down along the anterior left atrium (LA) to the coronary sinus ostium, where it combined with another signal that had come down along the RA septum. This combined signal travelled through the posterior LA via the LA-coronary sinus connection to the site just anterior to the isolated right pulmonary vein carina, entering the posterior interatrial connection. It then broke out to the mid-posterior RA through the connection.
