# Supplementary figures and images for: A macroreentrant biatrial tachycardia via the posterior interatrial connection emerged after extensive catheter ablation for paroxysmal atrial fibrillation
Source: HeartRhythm Case Rep. 2022 Oct 8;8(12):856–8. doi: 10.1016/j.hrcr.2022.10.009 (PMC9811108; doi:10.1016/j.hrcr.2022.10.009)

## Slide 1
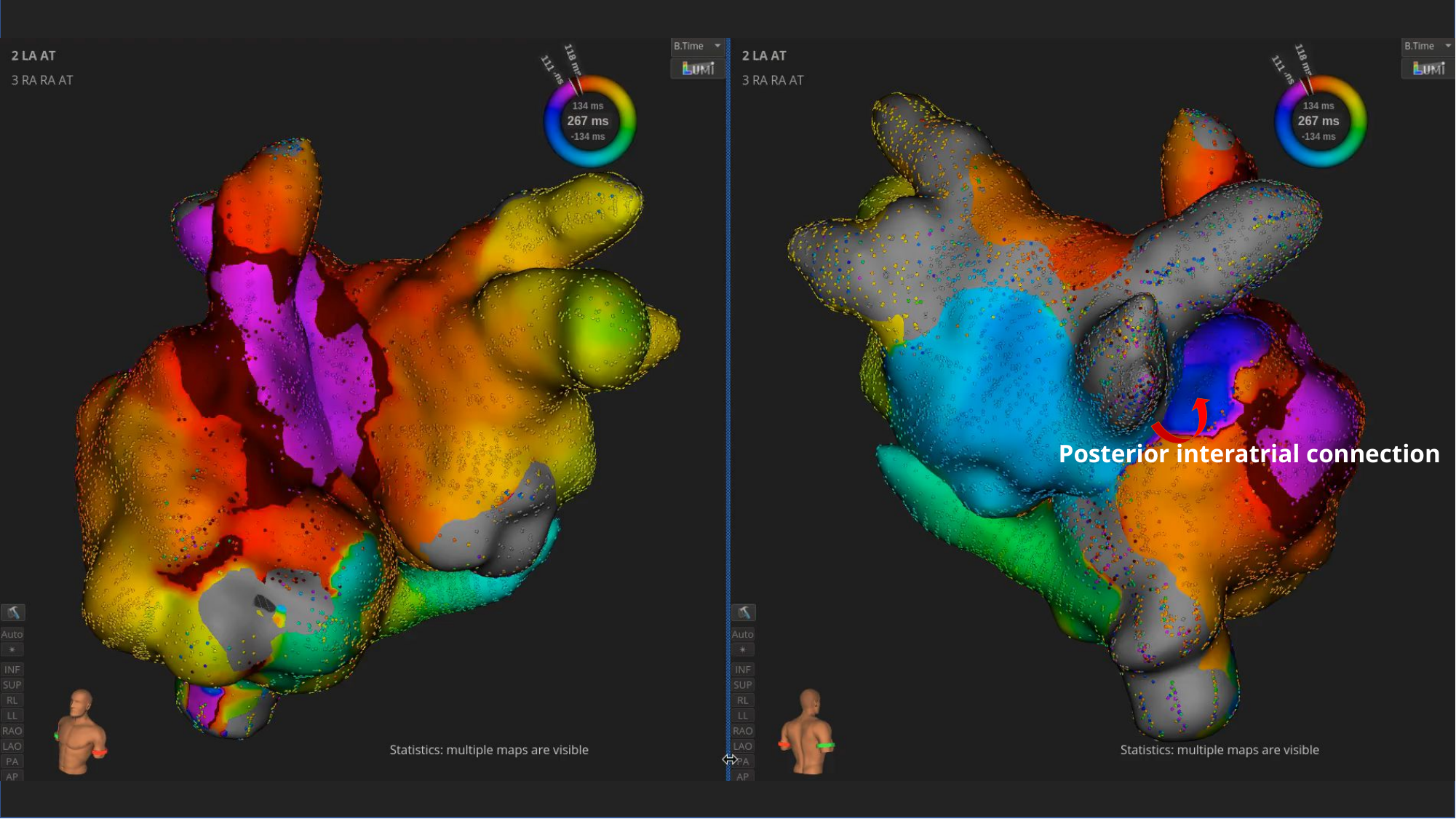

#
Posterior interatrial connection

Supplement: Supplemental Movie Legend — A high-resolution activation map of the atrial tachycardia demonstrates a centrifugal activation pattern in the right atrium (RA), with the earliest site at the mid-posterior RA. The signal traversed the RA at the antero-septum and mid-septum and was conducted to just outside the anterior right superior pulmonary vein isolation line through the Bachmann bundle. It then moved down along the anterior left atrium (LA) to the coronary sinus ostium, where it combined with another signal that had come down along the RA septum. This combined signal travelled through the posterior LA via the LA-coronary sinus connection to the site just anterior to the isolated right pulmonary vein carina, entering the posterior interatrial connection. It then broke out to the mid-posterior RA through the connection. [file mmc1.pptx]
